# Dataset of Vietnamese student's learning habits during COVID-19

**DOI:** 10.1016/j.dib.2020.105682

**Published:** 2020-05-08

**Authors:** Tran Trung, Anh-Duc Hoang, Trung Tien Nguyen, Viet-Hung Dinh, Yen-Chi Nguyen, Hiep-Hung Pham

**Affiliations:** a Vietnam Academy for Ethnic Minorities, Hanoi 100000, Vietnam; b EdLab Asia Educational Research and Development Centre, Hanoi 100000, Vietnam; c Institute of Theoretical and Applied Research, Duy Tan University, Hanoi 100000, Vietnam; d University of Labour Social Affairs, Hanoi 100000, Vietnam; e Phu Xuan University, Hue, Vietnam

**Keywords:** Learning habits, School closure, Socioeconomic, Occupational Aspiration, COVID-19, Vietnam, Secondary school

## Abstract

A dataset was constructed to examine Vietnamese student's learning habits during the time schools were suspended due to the novel coronavirus - SARS-CoV-2 (COVID-19), in response to a call for interdisciplinary research on the potential effects of the coronavirus pandemic (Elsevier, 2020). The questionnaires were spread over a network of educational communities on Facebook from February 7 to February 28, 2020. Using the snowball sampling method, researchers delivered the survey to teachers and parents to provide formal consent before they forwarded it to their students and children. In order to measure the influence of students’ socioeconomic status and occupational aspirations on their learning habits during school closures, the survey included three major groups of questions: (1) Individual demographics, including family socioeconomic status, school type, and occupational aspirations; (2) Student's learning habits, including hours of learning before and during the period of school suspension, with and without other people's support; and (3) Students’ perceptions of their self-learning during the school closures. There was a total of 920 clicks on the survey link, but only 460 responses accompanied by consent forms were received. Non-credible answers (e.g., year of birth after 2009, more than 20 hours of learning per day) were eliminated. The final dataset included 420 valid observations.

Specifications Table

**Table:** 

Subject	Education, Secondary Education
Specific subject area	Learning analytics, Socioeconomic, Occupational orientation
Type of data	Table Figure Excel file Sav file
How data were acquired	Data was gathered using an online survey and converted into .xlsx format for formal analysis in SPSS v.20
Data format	Raw Analyzed
Parameters for data collection	The target population of the survey was students in Hanoi who are learning online due to the effect of COVID-19. Only Grade 6-12 students were selected as they can evaluate their learning activities, and have more explicit occupational aspirations. Only students who had parental approval could access the survey.
Description of data collection	The data was conducted through an online questionnaire, which was delivered to Grade 6-12 students in Hanoi using the snowball sampling method.
Data source location	Information was collected from secondary schools in Hanoi (Latitude 21°1′28.2"N, Longitude 105°50′28.21"E), Vietnam
Data accessibility	Repository name: Mendeley repository Data identification number: Direct URL to data: http://dx.doi.org/10.17632/2pzvmnb2km.3

## Value of the data

• The dataset will be useful for researchers who want to compare students’ habits in a normal situation and unusual situations such as a pandemic.
• The dataset will be valuable to researchers who want to examine relationships between socioeconomic status, occupational aspirations, and students’ learning habits.
• The dataset will be useful for researchers who want to conduct comparative studies on students’ learning habits in different countries.
• The results of this dataset also contribute to enhancing educational leaders’ and policymakers’ awareness of the effects of sudden changes in educational scenarios, so education systems may be better prepared for similar situations in the future.

## 1 Data Description

Students’ learning habits are not the same during the school year and holidays. While a decrease in students’ formal learning habits during holidays is seasonal and predictable [1], the adjustments in their learning habits during a sudden pandemic are still unresearched. The preparation of this dataset is a response to the call for inter-disciplinary research about the effects of the novel coronavirus pandemic [2]. As a country that dealt with the COVID-19 outbreak very early and productively, Vietnam is a notable case study of instantaneous and conspicuous collaboration between the government and society [3]. However, the shift in the educational system was unforeseen and caused significant side effects [4]. This dataset [5] focused on the learning habits of 420 secondary students (Grade 6-12) in Hanoi during the first two weeks of school closures due to COVID-19. The dataset includes three major groups of variables: (A) Individual demographics, including family socioeconomic status (SES), school type, and occupational aspirations; (B) Students’ learning habits, including hours of learning before and during the period of school suspension, with and without other people's support; and (C) Students’ perceptions of their self-learning during the school closures. In addition, we added a question to measure the integration of online lessons during this time with sustainability topics. Detailed descriptions of all variables, together with the questions for each variable, and descriptive tables and figures can be found in the Mendeley data repository [5].

Tables 1, 2, 3 and 4.

**Table 1:** Descriptive statistics of demographics and students’ learning habits

Learning hours		N	Mean	Std. Deviation	Std. Error	Max	95% Confidence Interval for Mean		Min
							Lower Bound	Upper Bound	
**A. Students’ demographic**									
Gender	Male	166	1.57	.699	.054	3	1.47	1.68	1
	Female	239	1.59	.704	.046	3	1.50	1.68	1
	Not public	15	1.47	.640	.165	3	1.11	1.82	1
	Total	420	1.58	.699	.034	3	1.51	1.64	1
Grade level	Secondary school	234	1.61	.687	.045	3	1.52	1.70	1
	High school	186	1.54	.714	.052	3	1.43	1.64	1
	Total	420	1.58	.699	.034	3	1.51	1.64	1
School type	Public school (normal)	186	1.50	.668	.049	3	1.40	1.60	1
	Public school (Gifted)	132	1.65	.741	.065	3	1.52	1.78	1
	Private school (normal)	94	1.63	.672	.069	3	1.49	1.77	1
	International school	8	1.50	.926	.327	3	.73	2.27	1
	Total	420	1.58	.699	.034	3	1.51	1.64	1
Siblings	One	38	1.53	.797	.129	3	1.26	1.79	1
	Two	247	1.60	.684	.044	3	1.52	1.69	1
	Three	57	1.51	.685	.091	3	1.33	1.69	1
	Four or more	78	1.56	.713	.081	3	1.40	1.72	1
	Total	420	1.58	.699	.034	3	1.51	1.64	1
Father's job	STEM-related	141	1.59	.687	.058	3	1.47	1.70	1
	Social Science	172	1.64	.724	.055	3	1.53	1.75	1
	Free	73	1.51	.710	.083	3	1.34	1.67	1
	Others	34	1.35	.544	.093	3	1.16	1.54	1
	Total	420	1.58	.699	.034	3	1.51	1.64	1
Mother's job	STEM-related	32	1.59	.712	.126	3	1.34	1.85	1
	Social Science	270	1.62	.715	.044	3	1.53	1.70	1
	Free	63	1.43	.665	.084	3	1.26	1.60	1
	Others	55	1.53	.634	.085	3	1.36	1.70	1
	Total	420	1.58	.699	.034	3	1.51	1.64	1
Family income	Less than 430 USD	62	1.52	.671	.085	3	1.35	1.69	1
	From 430 to under 860 USD	141	1.48	.628	.053	3	1.37	1.58	1
	From 860 to under 1,290 USD	97	1.60	.745	.076	3	1.45	1.75	1
	From 1,290 to under 1,720 USD	50	1.80	.700	.099	3	1.60	2.00	1
	From 1,720 to under 2,150 USD	30	1.70	.794	.145	3	1.40	2.00	1
	More than 2,150 USD	40	1.60	.744	.118	3	1.36	1.84	1
	Total	420	1.58	.699	.034	3	1.51	1.64	1
University Entrance Exam subject group	A (Math, Physics, Chemistry)	52	1.48	.641	.089	3	1.30	1.66	1
	A1 (Math, Physics, English)	64	1.84	.672	.084	3	1.68	2.01	1
	B (Math, Biology, Chemistry)	23	1.70	.559	.117	3	1.45	1.94	1
	C (Literature, History, Geography)	22	1.41	.734	.157	3	1.08	1.73	1
	D (Literature, Foreign Language, Mathematics)	187	1.55	.727	.053	3	1.44	1.65	1
	Other	72	1.50	.671	.079	3	1.34	1.66	1
	Total	420	1.58	.699	.034	3	1.51	1.64	1
Self-evaluation of Academic performance	Below Average	7	1.14	.378	.143	2	.79	1.49	1
	Average	109	1.41	.596	.057	3	1.30	1.53	1
	Good	251	1.62	.702	.044	3	1.53	1.70	1
	Excellent	53	1.77	.824	.113	3	1.55	2.00	1
	Total	420	1.58	.699	.034	3	1.51	1.64	1
English language proficiency	Below Average	35	1.43	.655	.111	3	1.20	1.65	1
	Average	135	1.46	.620	.053	3	1.35	1.56	1
	Good	191	1.62	.721	.052	3	1.52	1.73	1
	Excellent	59	1.78	.767	.100	3	1.58	1.98	1
	Total	420	1.58	.699	.034	3	1.51	1.64	1
**B. Students’ learning habits**									
Learning time before COVID-19	under 4h	312	1.38	.560	.032	3	1.32	1.44	1
	from 4 to 7h	93	2.09	.732	.076	3	1.94	2.24	1
	over 7h	15	2.53	.743	.192	3	2.12	2.94	1
	Total	420	1.58	.699	.034	3	1.51	1.64	1
Learning time during COVID-19	under 4h	229	1.08	.292	.019	3	1.04	1.12	1
	from 4 to 7h	140	1.12	.388	.033	3	1.06	1.19	1
	over 7h	51	1.39	.666	.093	3	1.20	1.58	1
	Total	420	1.13	.398	.019	3	1.10	1.17	1
Online learning time during COVID-19	under 4h	304	1.37	.593	.034	3	1.30	1.43	1
	from 4 to 7h	88	1.97	.535	.057	3	1.85	2.08	1
	over 7h	28	2.64	.731	.138	3	2.36	2.93	1
	Total	420	1.58	.699	.034	3	1.51	1.64	1
Learning time with instruction	under 4h	373	1.53	.666	.034	3	1.46	1.60	1
	from 4 to 7h	38	1.82	.834	.135	3	1.54	2.09	1
	over 7h	9	2.44	.726	.242	3	1.89	3.00	1
	Total	420	1.58	.699	.034	3	1.51	1.64	1

**Table 2:** Descriptive statistics of students’ perceptions of their self-learning during school closures

C. Students’ perception of self-learning during COVID-19	N	Range	Min	Max	Mean		Std. Deviation
					Statistic	Std. Error	
**Self-learning during school closure due to COVID-19 is necessary because…**							
I can ensure my learning progress	420	4	1	5	3.90	.047	.965
I can maintain my learning habits	420	4	1	5	3.88	.045	.926
My teachers show me it is necessary	420	4	1	5	3.66	.050	1.031
My parents show me it is necessary	420	4	1	5	3.73	.050	1.019
My siblings show me it is necessary	420	4	1	5	3.27	.055	1.125
My friends show me it is necessary	420	4	1	5	3.25	.054	1.113
**I consider my self-learning activities are effective because…**							
I have motivation for self-learning	420	4	1	5	3.44	.049	.998
I have good concentration skills	420	4	1	5	3.36	.047	.970
I have support from my family	420	4	1	5	3.35	.053	1.090
I have an effective learning environment	420	4	1	5	3.55	.050	1.034
I can define my daily learning objectives	420	4	1	5	3.44	.050	1.017
I have various learning resources	420	4	1	5	3.66	.048	.983
I communicate and collaborate with my friends about learning	420	4	1	5	3.21	.055	1.129

**Table 3:** Correlations among variables and students’ total learning hours during COVID-19

*Variables*	*Total Learning hours during COVID-19*				*P-valure*
	*Sum of Squares*	*df*	*Mean Square*	*F*	
**Students’ demographics**					
Gender	.204	2	.102	.209	.812
Grade level	.496	1	.496	1.017	.314
School type	2.124	3	.708	1.455	.226
Siblings	.546	3	.182	.371	.774
Father's job	2.758	3	.919	1.895	.061**
Mother's job	1.998	3	.666	1.368	.252
Family income	4.695	5	.939	1.945	.086
University Entrance Exam subject group	24.148	2	12.074	4.208	.018***
Self-evaluation of Academic performance	6.717	3	2.239	4.708	.002***
English language proficiency	5.470	3	1.823	3.810	.014***
Learning hour before COVID-19	50.145	2	25.072	67.708	.000***
**Students’ perceptions about the necessity of learning during COVID-19**					
I can ensure my learning progress	3.360	4	.840	1.733	.061**
I can maintain my learning habits	11.884	4	2.971	6.399	.001***
My teachers show me it is necessary	2.879	4	.720	1.481	.207
My parents show me it is necessary	5.135	4	1.284	2.672	.032***
My siblings show me it is necessary	3.865	4	.966	1.998	.094
My friends show me it is necessary	3.121	4	.780	1.607	.171
**Students’ perception about factors that support learning during COVID-19**					
I have motivation for self-learning	20.711	4	5.178	11.687	.000***
I have good concentration skills	13.668	4	3.417	7.428	.000***
I have support from my family	6.083	4	1.521	3.180	.014***
I have an effective learning environment	12.054	4	3.013	6.496	.000***
I can define my daily learning objectives	21.514	4	5.378	12.194	.000***
I have various learning resources	12.963	4	3.241	7.019	.000***
I communicate and collaborate with my friends about learning	6.035	4	1.509	3.154	.014***

**Table 4:** Integration of online sessions with sustainability topics

	N	Range	Min	Max	Mean		Std. Deviation
					Statistic	Std. Error	
General Preventive Health care	420	4	1	5	3.85	.048	.985
Coronaviruses	420	4	1	5	3.93	.047	.959
Sustainable Environment Development	420	4	1	5	3.58	.049	0.995
Sustainable Society Development	420	4	1	5	3.49	.050	1.033
E-learning tools and techniques	420	4	1	5	3.35	.053	1.081

## 2 Experimental Design, Materials, and Methods

The survey was conducted between February 7 and February 28, 2020, the first three weeks of nationwide school closures due to COVID-19. Initially, online questionnaires were delivered to parents and teachers who were active in various educational forums on Facebook. Thereafter, it was spread by parents’ and teachers’ referrals. Parents or teachers were required to complete the consent form before forwarding the URL to the student. A total of 460 responses were received, but only 420 valid observations were accepted for further analysis, due to the elimination of obviously invalid answers (e.g. more than 20 hours of learning per day).

Overall, the influence of SES and students’ occupational aspirations on their learning habits during COVID-19 was examined using ordinary least squares (OLS) regression:

B∼β0+β1*A+β2*C+u

Theoretically, the survey was designed based on prior literature on transformative learning, with the focus on socioeconomic differences. Variables in group A related to students’ demographics, including SES factors and students’ self-evaluated competencies. Scholars have pointed out that SES factors such as monthly family income, parents’ occupations, number of siblings, school type, and grade level have significant influences on students’ learning habits [6,7]. This study complements the conventional notion of SES with additional variables about students’ competencies. Specifically in the case of Vietnam, we added subjects for university entrance, which demonstrate students’ occupational aspirations, and English, which is a crucial competency in today's world.

Variables in group B measured students’ learning habits by their learning hours per day [8]. In particular, students were asked their total hours of self-learning before and during COVID-19. With regard to the total number of learning hours during COVID-19, there were sub-questions about the total hours of off-line and online study modes, as well as the total hours of learning with instruction or without instruction from other people.

Variables in group C were mainly designed for this specific data collection. All items in this section were measured using a five-point Likert scale (1: Totally Disagree, 5: Totally Agree). First, we examined students’ perceptions on the necessity for self-learning during COVID-19. According to the literature on transformative learning, students’ learning practices are influenced by their beliefs about learning and influences from teachers, parents, and peers [9]. Thus, we constructed the variable of “students’ necessity for self-learning” using the following items: (i) to ensure my learning progress; (ii) to maintain my learning habits; (iii) influenced by teachers; (iv) influenced by parents; (v) influenced by siblings; (vi) influenced by friends. Second, we measured students’ self-reports on factors that influence self-learning effectiveness. This variable consisted of different physical factors (the availability of learning resources [10], learning space [11]), psychological factors (self-motivation [12], family support [13]), and behavioural factors (concentration, goal setting [14], communication and peer collaboration [15]).

In addition, with regard to the unique context of school closures due to COVID-19, we measured the integration of students’ online lessons with sustainability topics. Students were asked whether they were taught any of those topics or not: (i) General Preventive Health care; (ii) Coronaviruses; (iii) Sustainable Environment Development; (iv) Sustainable Society Development; (v) E-learning tools and techniques.

## Appendix Supplementary materials

Image, application 1
